# Amphetamines: a current epidemic

**DOI:** 10.3389/fpsyt.2025.1460341

**Published:** 2025-03-25

**Authors:** Norman Miller

**Affiliations:** Medical College of Georgia, Augusta University, Augusta, GA, United States

**Keywords:** amphetamine, ADHD, addiction, efficacy, long-term studies, misdiagnosis, overprescription, controlled substance act

## Introduction

The DSM-5 describes amphetamine stimulants as substances with a substituted-phenylethylamine structure (1). Amphetamines have a nearly identical structural makeup to methamphetamine and differ only by a single methylation. These stimulants are commonly prescribed to treat conditions such as obesity, ADHD, narcolepsy, and binge-eating disorder. Amphetamines are currently classified as Schedule-II drugs, meaning that they possess medically acceptable uses, but also have a high potential for abuse as well as severe psychological or physical dependence (2). The U.S. Centers for Disease Control and Prevention (CDC) explains that there is no singular test that can diagnose ADHD, and that there are many overlapping symptoms between ADHD and other disorders (3). Due to the risk of misdiagnosis and the limited amount of medical research into amphetamine addiction and long-term efficacy, practitioners should consider seeking additional educational resources before diagnosing patients with ADHD and prescribing daily, long-term use of amphetamines.

## Amphetamines: a present day opioid crisis

There is currently a high volume of regular amphetamine usage within the United States adult population (4). In 2022, an additional 1 million U.S. children aged 3-17 years received an ADHD diagnosis compared to 2016. The U.S. alone accounts for less than 5% of the world’s population, however, it represents 83.1% of the global volume of ADHD medications (4, 5). The prescription rate of amphetamines has drastically risen within the past decade, trending similarly to the spike in production and distribution of hydrocodone and oxycodone during the opioid crisis (6). Amphetamine production in 2006 was 7,943,299 grams and by 2021 it had reached over triple that amount, with the production quota by the FDA being set at 25,274,818 grams (7, 8). Between 2021 and 2022 alone, amphetamine distribution in states affected by the opioid epidemic were still continuing to rise, despite hydrocodone and oxycodone distribution significantly lowering (7, 8). This rise in amphetamine distribution may be due to addictive use, diversion, overdiagnosis, or other factors.

### Overdiagnosis and overprescription

The overdiagnosis and overprescription of amphetamines have reached an unprecedented national level (9). Amphetamine use has increased by more than double the amount from 2006 to 2016 (9). Additionally, in 2018, “among illicit substance users in the past year (53.2 million), more than 5 million 12 years or older had misused prescription stimulants” (9). Jeffrey A. Lieberman, former president of the American Psychiatric Association, highlighted this issue, noting that “the problem is not so much that we have a shortage of medication, but instead an overdiagnosis of the condition. There is no way that ADHD, as reflected by prescriptions for psychostimulants, can be multiples in frequency to what they are in western Europe and in other parts of the world.” (10) U.S. expenditures for ADHD medication have increased 594% between 1994 and 2003, with commercial insurance plans reportedly spending more per patient on medications intended for ADHD than for asthma, heart disease, hypertension, and dyslipidemia combined (4). The DSM-5 clearly notes that other medical and psychiatric disorders must be excluded prior to making a diagnosis of ADHD (1). Additionally, the DSM-5 states that “manifestations of the disorder must be present in more than one setting” and explains that “confirmation of substantial symptoms across settings typically can not be done accurately without consulting informants who have seen the individual in those settings.” (1) The confirmation of these symptoms may prove difficult in telemedicine environments. A virtual environment may make it difficult to fully observe behavioral indicators of ADHD such as fidgeting, impulsivity, or difficulty maintaining focus. This may result in a clinician misinterpreting symptoms that may also be present from a comorbid disorder.

### Pharmaceutical companies

The push for amphetamines as a regular treatment for an ADHD diagnosis can be traced back to the 1990s (11). As a result of the 1971 Psychotropic Convention, there was a decades-long resistance against direct-to-consumer advertising of controlled substances (12). Article 10 of the 1971 Convention requires that each party shall, subject to its constitutional limitations, prohibit the advertisement of psychotropic substances to the public. One major argument against direct-to-consumer advertising is that this advertising may omit important information surrounding the drugs.

Pharmaceutical companies began to push back, resulting in the relaxation of federal regulation regarding direct-to-consumer advertising of prescription drugs (13, 14). The pharmaceutical companies behind amphetamines then began spending millions of dollars in advertisements (15). Profit-based pharmaceutical companies clearly recognize the financial potential behind addictive drugs, as first demonstrated by the opioid epidemic as well as the tobacco industry, and now appearing again with the rise in amphetamine use.

The opioid epidemic during the late 2000s and early 2010s was partially a result of pharmaceutical companies exploiting the lucrative nature of addictive drugs (16, 17). The risk of addiction from opioid use was severely misrepresented. In fact, opioids were promoted in some cases for common conditions for which opioids are more likely to harm than help, such as low-back pain and fibromyalgia. Addiction involves a loss of control of use, meaning that many pharmaceutical companies benefit from the increase in demand of these drugs as well as their use outside of the prescribed amount and off-label uses. As a result, record levels of addiction were met on an individual level, while record sales and profits from opioids were met at a pharmaceutical level (16, 18). Pharmaceutical companies have also irrigated the channels running through every corner of the ADHD ecosystem, feeding into researchers, patient advocacy groups, celebrity spokespeople, advertisers, and more (19). Amphetamines proved to be financially successful for these pharmaceutical companies, with sales and the number of prescriptions rising within the decade preceding their introduction into the medical field (20). According to FDA manufacturer surveys, by 1962, US production reached an estimated 80000 kg of amphetamine salts (20). During the following years of the 1960s, FDA estimates of amphetamine production would grow little beyond 8 billion 10-mg doses, implying that consumption of the drug had already reached saturation levels in 1962 (20).

### FDA

The 1997 clarification by the FDA broadcast regulations allowed for Schedule II drugs to be advertised directly to consumers (13). However, the FDA has cited every major ADHD drug company for false and misleading advertising since 2000 (21). These citations for false and misleading advertising continued for two decades, with no significant attempt to limit the practice. There are currently no FDA approved pharmacological treatments to treat amphetamine addiction, which adds to the concern of the potential of an amphetamine epidemic (15). A similar situation can be seen when examining the FDA’s actions surrounding opioids. Opioids were first FDA approved in 1995, although in its 2001 rewording, the Food, Drug, and Cosmetic Act was ignored (22). In 1995, opioids were labeled as safe for “short-term” use. However, their usage was expanded again in 2001 to be effective for “daily, around-the-clock, long-term … treatment.” (22)

### Patients, hospital visits, and overdose

The COVID-19 pandemic presented statistics surrounding amphetamines: between 2020 and 2021, there was an 8% increase in individuals within the United States filling amphetamine prescriptions (23). There is also the issue of diversion. One common misconception that may lead to the diversion of amphetamines is that stimulants improve academic success and focus (24). Despite stimulant use being associated with short-term improvements on cognitive tasks, prolonged use has not been found to be associated with long-term improvements in academic achievements when compared with baseline performance (25).

Within the past decade, there has been an apparent number of amphetamine-related hospitalizations within the United States population (26). A 2021 review showed that the rate of amphetamine-related hospitalization increased from 27 to 69 per 100,000 population between 2003 and 2014 (27). Another study reported similar findings, with only a brief period of decline reported between 2005 to 2008 (26).

### Differential diagnoses

Some of the most common symptoms of ADHD include difficulty sustaining mental effort or cognitivity, impulsivity, alertness, fidgetiness, restlessness, inattention, and hyperactivity (1). The presentation of these symptoms may manifest in and mimic many different mental disorders aside from singularly ADHD itself (28). Due to this, it is not uncommon for symptoms often seen in ADHD to be better explained by another mental disorder listed in the DSM-5 (29). One of the hallmark symptoms of ADHD is inattentiveness or a difficulty concentrating and focusing. Problems with inattention and concentration can be present in not just ADHD, but also generalized anxiety disorder, post traumatic stress disorder, major depressive disorder, bipolar disorder, disruptive mood dysregulation disorder, psychotic disorders, neurocognitive disorders, and even medication-induced symptoms of ADHD (1) (30). Similar overlaps between symptoms of ADHD and other mental disorders have the potential to cause confusion and result in the misdiagnosis of patients with ADHD when their symptoms are already the result of another pre-existing condition (31, 32). These misdiagnoses carry the risk of not treating the correct cause of the diagnosis and expose individuals to risk of addiction and toxicity.

## Addiction: stimulant use disorder

Addiction is defined as the preoccupation with the acquisition and compulsive usage of a drug in spite of adverse consequences, with a history of relapse, and without regard to return of adverse effects and consequences (33). Impaired concentration and attention, impaired control of use, physical and mental manifestation of dependence, mood changes, anxiety, social problems, and risky use or behavior are potential adverse effects of amphetamine use and misuse (34, 35). It is important to note that some adverse effects of amphetamine use are also symptoms of ADHD. Amphetamine use may also be accompanied by a risk of psychosis.

A study conducted by Moran and her team concluded that “amphetamine use was associated with a greater risk of psychosis than methylphenidate.” (36) There were 343 episodes of psychosis within the study (with an episode defined as a new diagnosis code for psychosis and a prescription for an antipsychotic medication) among the 221,846 patients in the matched population. 106 episodes (0.10%) of these episodes occurred within the 110,923 patients in the methylphenidate group while 237 episodes (0.21%) occurred within the 110,923 patients in the amphetamine group (36).

Intoxication and cessation of the drug frequently results in depression, paranoid delusion, psychosis, hallucinations, excessive body temperature, mood swings, suicidal ideation, and panic attacks (37). Physical symptoms that occur during amphetamine use can include cardiac arrhythmia, stroke, and even death in some cases (38).Individuals exposed to amphetamines and amphetamine-type substances can develop stimulant use disorder as rapidly as one week while using the drug. Stimulant use disorder is characterized by a pattern of amphetamine-type substance, cocaine, or other stimulant use that leads to clinically significant impairment or distress (1). When induced by amphetamine use, symptoms include amphetamine usage of larger amounts over a longer than intended time period, persistent desire or unsuccessful efforts to reduce usage, large amounts of time being spent to obtain the stimulant, craving, tolerance, and withdrawal (1).

## Studies on amphetamines

ADHD as presented in adults has only recently become the focus of widespread clinical attention. This is made evident as it was not included in two major U.S. psychiatric epidemiological surveys within the past two decades, the Epidemiological Catchment Area Study and the National Comorbidity Survey (39). One review of controlled and naturalistic studies used a 24-week threshold, in which they reviewed the long-term efficacy and safety of amphetamine, methylphenidate, and atomoxetine (40). The study found no randomized control trials with amphetamine that met their long-term criteria (duration of 24 weeks or more) (40).

When examining methylphenidate, a similar gap in literature was apparent. In 2015, the Cochrane Collaboration’s synthesis of all existing studies and bodies of evidence found 185 studies, all of which focused on children and adolescents rather than the adult population (41) (42). Of those 185 studies, the vast majority were short-term, with an average length of two months (19). The study summarized that little could be concluded about the benefits and harm of methylphenidate used for longer than six months, similar to amphetamines. Additionally, there is a dangerous lack of studies on amphetamines centered around safety or their addiction potential specifically.

Without long-term studies, researchers are reduced to open label and naturalistic long-term studies, which present their own set of biases and drawbacks (40). Another factor to consider is the fact that ADHD has received less research funding than other mental-health conditions over the years. This lack of funding is in spite of the fact that pharmaceutical companies have rapidly increased their advertising budgets surrounding these medications (43). Between 1996 and 2003 spending on direct-to-consumer advertising increased by 400%, from $791 million to $3.2 billion (43). Furthermore, in 2004, the amount spent on direct-to-consumer advertising increased to over $4 billion, another 23% increase from the year prior (43). In 2022, the National Institute of Health granted $78 million for the study of ADHD, compared to $655 million allocated for depression (19).

## Discussion

The United States is currently experiencing an amphetamine epidemic jointly fueled by pharmaceutical companies, an overprescribing of amphetamines, a lack of efficacy studies, misdiagnosis of ADHD at increasing rates, and a high public demand for amphetamines. While amphetamines have a high potential for addiction, the prescribed compulsive and addictive use of amphetamines can be in perfect legal compliance, although it is accompanied by severe adverse consequences. It is important to note that there is a significant lack of longitudinal studies regarding long-term efficacy or addiction in the area of prescription stimulants.

Additionally, a large amount of the information that is able to be gathered on this subject is accessed through various websites, and newspapers rather than peer-reviewed sources. It is highly suggested that future research closes this clear gap in medical research. It is necessary to understand that research specifically focused on the long-term effects and efficacy of amphetamines is a vital part of improving the issue of informed consent within the process of being prescribed amphetamines. Furthermore, it is recommended that due to the addictive nature of prescribed amphetamines, practitioners should take additional caution when diagnosing an individual with ADHD. A proper diagnosis takes time and multiple consultations, as the DSM-5 states that “confirmation of substantial symptoms across settings typically can not be done accurately without consulting informants who have seen the individual in those settings.” (1) Schedule II drugs have a high potential to be addictive when misused, especially without proper transparency and caution from a medical professional.
